# Passive leg raising can predict fluid responsiveness in patients placed on venovenous extracorporeal membrane oxygenation

**DOI:** 10.1186/cc10451

**Published:** 2011-09-18

**Authors:** Pierre-Grégoire Guinot, Elie Zogheib, Mathieu Detave, Mona Moubarak, Vincent Hubert, Louise Badoux, Eugénie Bernard, Patricia Besserve, Thierry Caus, Hervé Dupont

**Affiliations:** 1 Department of Anesthesiology and Critical Care, Amiens University Hospital, Place Victor Pauchet, Amiens, F-80054, France; 2 Department of Cardiac Surgery, Amiens University Hospital, Place Victor Pauchet, Amiens, F-80054, France; 3 INSERM, ERI12, Jules Verne University of Picardy, 12 rue des Louvels, Amiens, F-80000, France

**Keywords:** acute respiratory distress syndrome, fluid responsiveness, passive leg raising, extracorporeal membrane oxygenation, venovenous

## Abstract

**Introduction:**

In ICUs, fluid administration is frequently used to treat hypovolaemia. Because volume expansion (VE) can worsen acute respiratory distress syndrome (ARDS) and volume overload must be avoided, predictive indicators of fluid responsiveness are needed. The purpose of this study was to determine whether passive leg raising (PLR) can be used to predict fluid responsiveness in patients with ARDS treated with venovenous extracorporeal membrane oxygenation (ECMO).

**Methods:**

We carried out a prospective study in a university hospital surgical ICU. All patients with ARDS treated with venovenous ECMO and exhibiting clinical and laboratory signs of hypovolaemia were enrolled. We measured PLR-induced changes in stroke volume (ΔPLRSV) and cardiac output (ΔPLRCO) using transthoracic echocardiography. We also assessed PLR-induced changes in ECMO pump flow (ΔPLRPO) and PLR-induced changes in ECMO pulse pressure (ΔPLRPP) as predictors of fluid responsiveness. Responders were defined by an increase in stroke volume (SV) > 15% after VE.

**Results:**

Twenty-five measurements were obtained from seventeen patients. In 52% of the measurements (*n *= 13), SV increased by > 15% after VE (responders). The patients' clinical characteristics appeared to be similar between responders and nonresponders. In the responder group, PLR significantly increased SV, cardiac output and pump flow (*P *< 0.001). ΔPLRSV values were correlated with VE-induced SV variations (*r*^2 ^= 0.72, *P *= 0.0001). A 10% increased ΔPLRSV predicted fluid responsiveness with an area under the receiver operating characteristic curve (AUC) of 0.88 ± 0.07 (95% confidence interval (CI_95_): 0.69 to 0.97; *P *< 0.0001), 62% sensitivity and 92% specificity. On the basis of AUCs of 0.62 ± 0.11 (CI_95_: 0.4 to 0.8; *P *= 0.31) and 0.53 ± 0.12 (CI_95_: 0.32 to 0.73, *P *= 0.79), respectively, ΔPLRPP and ΔPLRPO did not predict fluid responsiveness.

**Conclusions:**

In patients treated with venovenous ECMO, a > 10% ΔPLRSV may predict fluid responsiveness. ΔPLRPP and ΔPLRPO cannot predict fluid responsiveness.

## Introduction

In ICUs, fluid administration is frequently used to treat hypovolaemia to enhance cardiac function by increasing preload. Many studies have demonstrated that fluid responsiveness can be predicted by using respiratory derivative indices (pulse pressure variation (ΔrespPP), stroke volume (SV) variation (ΔrespSV) and aortic velocity-time integral variation (ΔrespVTIAo)) [1-5]. From a clinical perspective, owing to altered alveolar capillary membrane permeability, fluid management is critical to the outcomes of ARDS patients [6,7]. In ARDS, dynamic indices predictive of fluid responsiveness present limitations related to the effects of the cardiopulmonary disease on heart-lung interactions (right-sided heart failure, pulmonary hypertension and protective ventilation) [8-12]. In addition, as fluid overload can be harmful, indices using passive leg raising (PLR) have been validated [13-17]. By shifting blood from the lower limbs and splanchnic compartment, PLR is a safe, reversible manoeuvre that mimics fluid expansion [16]. In adult patients with refractory ARDS, despite ventilatory optimisation by means of routine therapies (protective mechanical ventilation, prone position and nitric oxide) [18,19], the use of respiratory assistance such as venovenous extracorporeal membrane oxygenation (ECMO) ensures oxygenation and decarboxylation [20-22]. Venovenous ECMO consists of a circuit supplied by a centrifugal pump without a venous reservoir. Venous return and pump venous injection are preload- and postload-dependent processes that run in parallel to the human right-sided circulation and may interfere with it [23-25]. In hypovolaemic patients, PLR prediction of fluid responsiveness is unclear. Blood transfer induced by PLR may be modified by the preload dependence of the ECMO. Because patients supported by ECMO are the frailest ARDS patients, because they present with several interrelated diseases that limit the use of respiratory dynamic criteria, and because fluid therapy can affect outcomes, it is necessary to validate additional manoeuvres such as PLR that may better discriminate responders from nonresponders.

The main goal of this study was to answer the following question: Can PLR be used to predict fluid responsiveness in ARDS patients placed on venovenous ECMO? As ECMO pump flow (PO) is a preload-dependent process, we assumed that changes in PO between baseline and PLR (ΔPLRPO) could reflect a preload-dependent condition. Thus, we analysed the predictive value of ΔPLRPO.

## Materials and methods

### Patients

We conducted a prospective, observational study at the Amiens Sud University Hospital surgical ICU over a period of 13 months (from November 2009 to December 2010). ARDS patients treated with venovenous ECMO for whom the intensivist recommended volume expansion (VE) were enrolled in the study. Patients with poor cardiac echogenicity were not included. All patients had been sedated and paralysed with continuous infusion of midazolam, sufentanil and cisatracurium. All patients underwent invasive arterial pressure monitoring, central venous catheterisation and echocardiography several times daily because of their various diseases.

This study was approved by the Institutional Review Board (IRB) for human subjects at our hospital. Informed consent was waived because the IRB considered the protocol to be part of routine clinical practice.

### Extracorporeal membrane oxygenation

The ECMO circuit consists of an inflow venous line inserted into the right femoral vein and advanced through the inferior vena cava close to the right atrium, an outflow venous line inserted into the right internal jugular vein as far as the right atrium, a ROTAFLOW centrifugal pump (MAQUET GmbH & Co. AG, Rastatt, Germany) and a QUADROX PLS oxygenator (MAQUET GmbH & Co. AG). Inflow and outflow cannula sizes are reported in Table 1.

**Table 1:** Main patient characteristics at the time of inclusion^a^

Patient	Gender	ARDS aetiology	Outflow/inflow cannula size (French)	PaO_2_/FiO_2 _ratio at ECMO assistance	Time from assistance with ECMO (days)	Tidal volume (mL/kg)	Respiratory compliance (mL/cmH_2_O)	Norepinephrine	Epinephrine or dobutamine	Acute cor pulmonale
1	M	Community-acquired pneumonia	18/25	42	2	4	17.6	Yes	No	Yes
					8	6	34.3	Yes	No	Yes
2	F	H1N1	18/22	45	3	2.6	9.6	No	Yes	Yes
3	M	H1N1	18/23	42	5	4.1	16	Yes	No	Yes
4	M	Postoperative bacterial pneumonia	18/25	37	2	5.4	26.9	Yes	Yes	Yes
					12	7.1	41.8	Yes	Yes	Yes
5	M	Peritonitis	18/25	60	4	4.4	24.6	Yes	No	No
					7	5.1	28.7	Yes	No	No
6	F	H1N1	18/24	45	5	3.7	14.7	yes	Yes	Yes
7	M	Postoperative bacterial pneumonia	18/25	38	6	6	23.5	Yes	Yes	Yes
					6	3.8	10.4	Yes	Yes	Yes
8	F	Community-acquired pneumonia	18/25	50	7	5.8	21.3	No	No	No
9	F	H1N1	16/24	39	4	3.6	15	Yes	Yes	Yes
					12	5	27.3	Yes	No	No
10	M	Chest trauma	18/25	38	5	3.4	20.8	Yes	No	Yes
					11	4.8	23.8	No	No	No
11	F	Community-acquired pneumonia	16/25	58	11	5.6	16.3	No	No	No
					15	8	24.4	No	No	Yes
12	M	Postoperative bacterial pneumonia	16/25	45	4	2.9	12.2	Yes	Yes	Yes
					8	4.6	15.9	Yes	No	Yes
13	M	Community-acquired pneumonia	18/25	87	5	2.8	11.7	No	Yes	Yes
14	M	Bronchopleural fistula	18/25	52	10	2.9	12.2	Yes	Yes	Yes
15	M	Postoperative bacterial pneumonia	18/25	44	5	3.3	16.7	No	Yes	Yes
16	M	H1N1	18/24	50	6	5.7	38.2	Yes	No	No
17	F	Community-acquired pneumonia	16/23	46	3	3.4	16.7	Yes	No	Yes

^a^ARDS, acute respiratory distress syndrome; PaO_2_/FiO_2 _ratio, ratio of partial pressure of arterial oxygen to fraction of inspired oxygen; ECMO, extracorporeal membrane oxygenation; M, male; F, female. Patients 1, 4, 5, 7, 9, 10, 11 and 12 underwent two sets of measurements.

### Measurements

The following clinical features were measured: age; gender; weight; surgical, medical and/or clinical problems; and the main diagnosis. Transthoracic echocardiography (TTE) was performed by a single physician using a Philips EnVisor Ultrasound System (Philips Medical Systems, Suresnes, France). The diameter of the aortic annulus (AoD) was measured using a long-axis parasternal view at patient inclusion. Aortic area (Aa) was calculated by using the equation Aa (in cm^2^) = (π × AoD^2^)/4. The aortic velocity-time integral (VTIAo) ratio was measured using pulsed Doppler ultrasonography with a five-chamber apical view. SV was calculated by using the equation SV (in mL) = VTIAo × Aa. Cardiac output (CO) was calculated using the formula CO (in mL/minute) = SV × heart rate (HR). Mean echocardiographic values were calculated from five measurements (regardless of the respiratory cycle) and analysed *a posteriori*. The reproducibility of aortic area and VTIAo measurements were tested before the study. Intraindividual and interindividual reproducibility were calculated from the mean value of three of ten patients. The mean (± standard deviation) intraindividual and interindividual reproducibility values were 4.4 ± 3.9% and 4.4 ± 3.2%, respectively. The ECMO PO was measured on the monitor screen by a physician at the different steps of the study. Maximum and minimum pump flow (PO_max _and PO_min_, respectively) were recorded for one minute. Mean PO was calculated as PO (in mL/minute) = (PO_max _+ PO_min_)/2. PO variation was called the pulse index (PI) and calculated as PI = (PO_max _- PO_min_)/(PO_max _+ PO_min_/2) × 100. The PO/rotation per minute (RPM) ratio (expressed in mL/rotation/minute) was calculated as (PO/RPM) × 1,000. Central venous pressure (CVP) and blood pressure were measured with a transducer zeroed at the level of the midaxillary line. ΔrespPP was measured on frozen waveforms on the monitor by calculating ΔrespPP = (PP_max _- PP_min_)/[(PP_max _+ PP_min_)/2)] × 100.

### Study protocol

Systolic arterial pressure (SAP), median arterial pressure (MAP), diastolic arterial pressure (DAP), ΔrespPP, CVP, CO, VTIAo and HR were recorded at baseline with the patient in a semirecumbent position (45° angle). Automatic bed raising from this position raised the patient's lower limbs to a 45° angle while the patient's trunk was lowered from a semirecumbent to supine position [11]. A second set of SAP, MAP, DAP, HR, CVP, PO, ΔrespPP and VTIAo measurements was recorded when VTIAo plateaued at its highest value. The patient was then returned to the initial semirecumbent position, and VE was initiated with 500 mL of saline for 15 minutes. A third set of measurements was recorded after VE. The ventilator settings, drugs and ECMO RPM were maintained at constant levels throughout the study period.

### Statistical analysis

SV measured before and after VE was used to distinguish responders from nonresponders with changes in SV of > 15% and < 15%, respectively [7,9,12]. The results are expressed as medians (25th to 75th interquartile ranges). Changes in haemodynamic variables were compared between responders and nonresponders before PLR and VE using the nonparametric Mann-Whitney *U *test. For the overall population and for each subgroup (responders and nonresponders), the nonparametric Wilcoxon rank-sum test was used to assess the statistical significance of changes in PLR-induced haemodynamic parameters or VE and to compare the pre-PLR values of the variables measured at baseline (HR, SAP, DAP, MAP, SV, VTIAo, PO and PI). Spearman's rank correlation coefficient was used to test linear correlations. A receiver operating characteristic curve (ROC) and its corresponding positive and negative likelihood ratios were generated for PLR-induced changes in SV (ΔPLRSV), CO (ΔPLRCO) and PO (ΔPLRPO) [26]. Area under the ROC (AUC) values for ΔPLRSV and ΔPLRCO were compared. Differences with a *P *value < 0.05 were considered statistically significant. Statistical analysis was performed using IBM SPSS Statistics 18 software (IBM Corp., Armonk, NY, USA) and MedCalc 8.1.0.0 software (Mariakerke, Belgium).

## Results

Twenty-five measurements were obtained from seventeen patients. PLR manoeuvres and VE were performed twice on eight patients on different days and under different respiratory and haemodynamic conditions. The patients had acute circulatory failure associated with septic shock and primary or secondary ARDS. The most common cause of heart failure was acute cor pulmonale, defined on the basis of echocardiographic criteria [27]. The patient characteristics are reported in Table 1. All VEs were performed on the basis of the following criteria: arterial hypotension (SAP < 90 mmHg and/or MAP < 70 mmHg) (*n *= 12), oligoanuria (urine output < 0.5 mL/kg/hour or < 20 mL/hour) (*n *= 9), skin mottling and/or leg coldness (*n *= 4).

**Figure 1:**
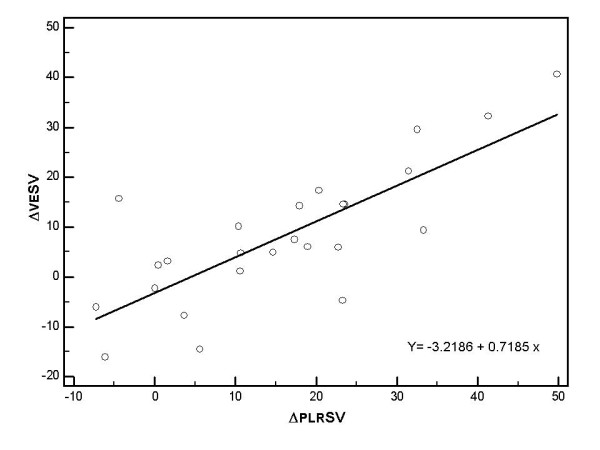
**Relation between changes in stroke volume induced by PLR (ΔPLRSV) and changes in stroke volume induced by volume expansion (ΔVESV)**.

Of the 25 patients who underwent VE, 13 (52%) were responders: SV increased by > 15%. At baseline, the haemodynamic and echocardiographic parameters were similar in both groups. None of these parameters were predictive of fluid responsiveness. Tables 2 and 3 present the haemodynamic, echocardiographic and PO parameters. SV and CO increases during PLR and after VE were correlated (respectively, *r*^2 ^= 0.72, *P *= 0.0001 (Figure 1); *r*^2 ^= 0.70, *P *= 0.0001). This correlation was not observed for PO (*r*^2 ^= 0.07, *P *= 0.79).

**Table 2:** Comparison of haemodynamic parameters between responders and nonresponders^a^

Parameters	Responders (*n *= 13)	Nonresponders (*n *= 12)	*P *values
HR (bpm)			
Baseline	95 (82 to 106)	95 (81 to 104)	0.95
PLR	95 (76 to 104)	91 (77 to 106)	0.95
Volume expansion	93 (78 to 105)	96 (79 to 106)	0.72
SAP (mmHg)			
Baseline	108 (99 to 121)	105 (92 to 115)	0.47
PLR	117 (93 to 121)	107 (86 to 120)	0.46
Volume expansion	120 (111 to 130)^b,c^	107 (99 to 126)	0.2
DAP (mmHg)			
Baseline	59 (49 to 71)	57 (46 to 70)	0.62
PLR	61 (44 to 67)	61 (44 to 70)	1
Volume expansion	67 (49 to 72)^b, c^	56 (50 to 70)	0.51
MAP (mmHg)			
Baseline	77 (65 to 84)	75 (62 to 83)	0.62
PLR	77 (61 to 84)	72 (58 to 84)	0.64
Volume expansion	83 (71 to 90)^b, c^	71 (67 to 87)	0.27
CVP (mmHg)			
Baseline	7 (5 to 13)	11 (8 to 15)	0.08
PLR	12 (8 to 14)^b^	12 (10 to 13)	0.68
Volume expansion	12 (10 to 15)^b^	13 (10 to 17)^b^	0.46
ΔrespPP (%)			
Baseline	6 (4 to 8)	7 (4 to 10)	0.59
PLR	6 (4 to 7)	9 (5 to 11)	0.14
Volume expansion	5 (4 to 8)	7 (5 to 10)	0.11
VTI (cm/second)			
Baseline	20 (15.2 to 25)	16.1 (13.7 to 21.6)	0.3
PLR	24.7 (17.7 to 28.3)^b^	15.8 (12.9 to 22.3)	0.03
Volume expansion	25.4 (19.1 to 30)^b, c^	16 (14 to 21.4)	< 0.001
SV (mL)			
Baseline	72 (50 to 88)	55 (49 to 84)	0.7
PLR	86 (59 to 99)^b^	58 (48 to 85)	0.1
Volume expansion	89 (66 to 109)^b, c^	60 (48 to 82)	0.02
CO (L/minute)			
Baseline	5.8 (4 to 8.3)	5.6 (4.3 to 7.8)	0.96
PLR	5.9 (4.7 to 9.6)^b^	5.8 (4 to 6.6)	0.27
Volume expansion	7.5 (5.2 to 10.1)^b, c^	5.2 (4.4 to 7.7)	0.03

^a^PLR, passive leg raising; HR, heart rate; SAP, systolic arterial pressure; DAP, diastolic arterial pressure; MAP, median arterial pressure; CVP, central venous pressure; ΔrespPP, respiratory pulse pressure variation; VTI, velocity time integral of aortic blood flow; SV, stroke volume; CO, cardiac output. ^b^*P *< 0.05 vs baseline; ^c^*P *< 0.05 vs PLR. Values are medians (25th to 75th interquartile ranges).

**Table 3:** Comparison of extracorporeal membrane oxygenation parameters between responders and nonresponders^a^

Parameters	Responders (*n *= 13)	Nonresponders (*n *= 12)	*P *values
PO (L/minute)			
Baseline	4.70 (3.45 to 4.99)	4.25 (3.49 to 5.02)	0.91
PLR	4.76 (3.24 to 5.05)	4.23 (3.5 to 5)	0.83
Volume expansion	4.81 (3.36 to 5.14)^b, c^	4.20 (3.57 to 5.09)	0.97
PI (%)			
Baseline	0.95 (0.59 to 1.9)	0.78 (0.48 to 1.20)	0.35
PLR	0.68 (0.4 to 1)^b^	0.69 (0.45 to 1)	0.78
Volume expansion	0.62 (0.6 to 1.3)^b, c^	0.91 (0.54 to 1.1)	0.05
RPM	3950 (3078 to 4004)	3838 (3010 to 3966)	0.96
PO/RPM ratio (mL/RPM)			
Baseline	1.24 (1.07 to 1.31)	1.23 (1.11 to 1.32)	0.83
PLR	1.26 (1.03 to 1.33)^b^	1.23 (1.13 to 1.32)	0.62
Volume expansion	1.27 (1.06 to 1.32)^b, c^	1.25 (1.13 to 1.32)	0.68
PO/CO ratio (%)			
Baseline	66 (60 to 91)	75 (56 to 93)	0.7
PLR	60 (51 to 77)^b^	73 (62 to 105)	0.05
Volume expansion	54 (49 to 69)^b, c^	72 (55 to 100)	0.05

^a^PLR, passive leg raising; PO, pump outflow; PI, pulse index; RPM, rotations per minute; CO, cardiac output. ^b^*P *< 0.05 vs baseline; ^c^*P *< 0.05 vs PLR. Values are medians (25th to 75th interquartile ranges).

**Figure 2:**
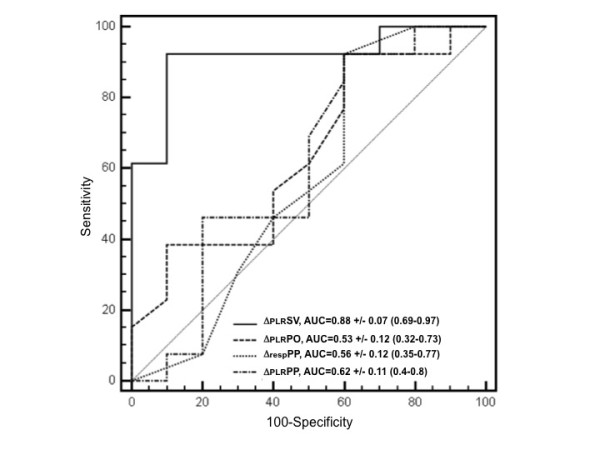
**Receiver operating characteristic curves discriminating responders and nonresponders to volume expansion**. ΔPLRSV, changes in stroke volume from baseline until after passive leg raising; ΔPLRPO, changes in pump outflow from baseline until after passive leg raising; ΔrespPP, respiratory variation of pulse pressure at baseline; ΔPLRPP, changes in pulse pressure from baseline until after passive leg raising.

In the whole population, the AUCs were 0.88 ± 0.07 for ΔPLRSV (95% confidence interval (CI_95_): 0.69 to 0.97; *P *< 0.0001) and 0.87 ± 0.07 for ΔPLRCO (CI_95_: 0.67 to 0.97; *P *< 0.0001) (Figure 2). The ΔPLRCO and ΔPLRSV AUCs were not different (*P *= 0.66). ΔPLRPO had poor sensitivity and specificity, as well as a poor AUC: 0.53 ± 0.12 (CI_95_: 0.32 to 0.73; *P *= 0.79) (Figure 2). The ΔrespPP AUC was 0.56 ± 0.12 (CI_95_: 0.35 to 0.77; *P *= 0.59) (Figure 2). The ΔPLRPP AUC was 0.62 ± 0.11 (CI_95_: 0.4 to 0.8; *P *= 0.31) (Figure 2). In ten patients, CVP increased by ≥ 2 mmHg (six responders and four nonresponders). Among these patients, the AUCs were 0.83 ± 0.13 for ΔPLRSV (CI_95_: 0.48 to 0.98; *P *= 0.012) and 0.83 ± 0.13 for ΔPLRCO (CI_95_: 0.48 to 0.98; *P *= 0.012). Table 4 reports the different threshold values of ΔPLRSV and ΔPLRCO.

**Table 4:** Accuracy of stroke volume and cardiac output changes after passive leg raising to predict fluid responsiveness^a^

Criteria	Sensitivity	Specificity	LR+	LR-	PPV	NPV
ΔPLRSV > 3%	92% (64 to 100)	67% (35 to 90)	2.7 (1.8 to 4.3)	0.12 (0.01 to 0.9)	75% (48 to 93)	89% (49 to 100)
ΔPLRSV > 5%	92% (64 to 100)	83% (52 to 98)	5.5 (4.1 to 7.7)	0.09 (0.01 to 0.9)	86% (57 to 98)	91% (59 to 100)
ΔPLRSV > 10%	62% (32 to 86)	92% (62 to 100)	7.3 (4.7 to 11.7)	0.42 (0.06 to 3.1)	89% (52 to 100)	69% (40 to 90)
ΔPLRSV > 15%	39% (14 to 68)	92% (62 to 100)	4.6 (2.3 to 9.4)	0.67 (0.1 to 4.6)	83% 36 to 100)	58% (34 to 80)
ΔPLRCO > 3%	92% (64 to 100)	67% (35 to 90)	2.8 (1.8 to 4.3)	0.12 (0.01 to 0.9)	75% (48 to 93)	89% (49 to 100)
ΔPLRCO > 5%	85% (46 to 95)	83% (52 to 98)	5 (3.6 to 7.2)	0.18 (0.03 to 1.1)	85% (55 to 98)	83% (52 to 98)
ΔPLRCO > 8%	69% (39 to 91)	83% (52 to 98)	4.1 (2.7 to 6.5)	0.37 (0.08 to 1.7)	82% (48 to 98)	71% (42 to 92)
ΔPLRCO > 12%	54% (25 to 81)	83% (52 to 98)	3.2 (1.8 to 5.7)	0.55 (0.1 to 2.2)	78% (38 to 98)	63% (35 to 85)

^a^LR+, positive likelihood ratio; LR-, negative likelihood ratio; PPV; positive predictive value; NPV, negative predictive value; ΔPLRSV, stroke volume increase from baseline and after passive leg raising; ΔPLRCO, increase of cardiac output increase from baseline and after passive leg raising. Data are raw numbers and CI_95 _ranges.

## Discussion

**Figure 3:**
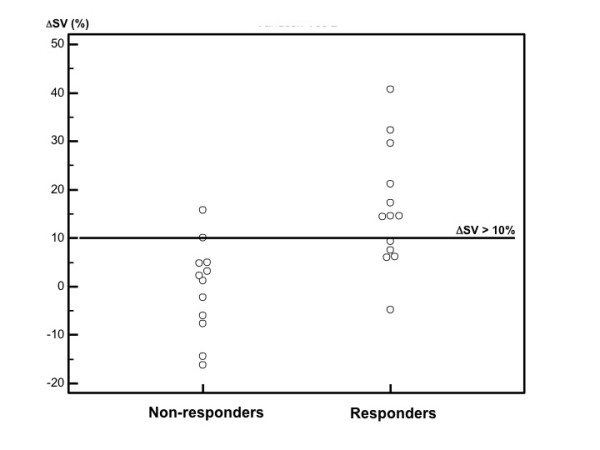
**Passive leg raising (PLR)-induced stroke volume (SV) increase in responders and nonresponders**. ΔSV, stroke volume increase between baseline and PLR expressed as a percentage.

Our study demonstrates that a 10% increase ΔPLRSV predicts a > 15% increase in SV after VE in ARDS patients placed on venovenous ECMO (Figure 3). ECMO is an efficient treatment for refractory ARDS [22]. No studies have assessed the usual dynamic parameters of preload reserve on ECMO-assisted patients, probably because of the complex interactions between the protective mechanical ventilation and ECMO system on heart-lung interaction. As previously shown in the context of ARDS without ECMO support, neither ΔrespPP nor ΔPLRPP predicts fluid responsiveness [8-10,12]. In two prospective studies, the poor predictive performance of ΔrespPP has been attributed to insufficient changes in transpulmonary and pleural pressure [10,12], which are related to protective ventilation and altered pulmonary compliance. In addition, ΔrespPP could reflect postload variation on right ventricular dysfunction and cannot be used as a predictor even in the presence of hypovolaemia [8,9,11]. Such clinical situations are frequent in the treatment of ECMO-assisted patients, which limit the use of such indices. ECMO patients with late ARDS were ventilated with 'ultraprotective' ventilation because of altered compliance and a high incidence of acute cor pulmonale (Table 1). As Δresp indices must be avoided because they fail to predict fluid responsiveness and fluid overload, fluid management may rely on a reversible and safe fluid challenge. Thus, we assessed predictive values of ΔPLR indices.

ΔPLRPP cannot predict fluid responsiveness, even among patients with a minimal increase in CVP of 2 mmHg. On the basis of physiological knowledge, the use of PP as a substitute for SV would assume constant arterial compliance. By increasing intrathoracic blood volume, PLR may also induce sympathetic activation; however, our patients were deeply sedated, and their HRs remained unchanged during PLR. In clinical practice, SV, PP and vascular tone can vary with the patient's haemodynamic conditions and can be altered by PLR [28], which may have been the case in our present study.

In this context, echocardiographic measurement of ΔPLRSV and ΔPLRCO may predict fluid responsiveness. The best threshold was 5% for ΔPLRSV, with 92% sensitivity (CI_95_: 64 to 100) and 83% specificity (CI_95_: 52 to 98), and 5% for ΔPLRCO, with 85% sensitivity (CI_95_: 46 to 95) and 83% specificity (CI_95_: 52 to 98) (Table 4). The lower sensitivity of ΔPLRCO may be explained by the fact that CO is the product of SV and HR. In some responders, the VE-induced SV increase was associated with decreased HR (nonsignificantly). Whereas sensitivity differed, the ΔPLRCO and ΔPLRSV AUCs were not statistically different. Taking into account echocardiography, interindividual reproducibility and the fact that we wanted to avoid volume overloading, a threshold of 10% for ΔPLRSV was proposed, with 92% specificity and 62% sensitivity (Table 4 and Figure 3).

Although ECMO modifies the right-sided heart load condition and interferes with pulmonary artery circulation [20,23,24], it does not prevent an increase in venous return, as also reflected by increased CVP and persistent fluid responsiveness during PLR and after VE with sensitivity and specificity percentages comparable to values usually recorded in the ICU without ECMO [13-15,17]. One assumption might be that, in hypovolaemic patients, fluid expansion may load the ECMO pump first and alter VE (and ΔPLR predictive values), as patients' SV may not increase. In four nonresponders, PO increased but SV also increased by > 10%. Moreover, ΔPLRSV were correlated with VE-induced SV variations, with values close to those recorded in the ICU among patients not being treated with ECMO [14]. Thus, in nonresponders, an increase in CVP without a change in CO and PO confirms an increase in right-sided preload and the fact that fluid expansion should not refill the ECMO system. Unlike Lakhal *et al. *[15], we found that the CVP measurement did not improve the accuracy of ΔPLR indices. CVP values should be analysed with caution, even if the mean CVP values increased with PLR and VE. Since the distal extremities of the ECMO canula and CVC are close, the CVP mean value may vary depending on measurement variations. In addition, this subgroup analysis was performed in only 10 patients.

Since venous return and pump venous injection are preload- and afterload-dependent processes, we assumed that ECMO parameters could demonstrate preload-dependent conditions. At baseline, the PO/RPM ratio, which may reflect PO adequacy to preload status, was not different between the two groups. PLR and VE increase PO and PO/RPM ratio and decreased PI only when the patient was preload-dependent, that is, only in responders (Table 3). Nevertheless, ΔPLRPO was not predictive of fluid responsiveness (Figure 2). Different mechanisms may explain this result. First, low variations in PO may have exposed ΔPLRPO to an insufficient signal-to-noise ratio that was distorted by our method of calculating PO. Second, the afterload dependency of ECMO may alter PO. As we did not investigate ECMO afterload, we cannot link an increase in PO to an increase in ECMO preload and/or a decrease in ECMO afterload. Indeed, the present results confirm the cardiac dependence of the venovenous ECMO circuit. The venovenous ECMO circuit acts as a cardiac preload sensor (more than a volume sensor). Nonetheless, for the reasons mentioned above, we have not demonstrated that ΔPLRPO is predictive of fluid responsiveness.

This study has a number of limitations. The small number of measurements may limit the interpretation of the results, but this number is comparable to the sample sizes of other published studies and the statistical significance is sufficient [17]. The ΔPLRSV cutoff is close to ultrasonic interindividual reproducibility. This threshold is comparable to values usually recorded in the ICU [17,29] and is more than twice the values of inter- and intraindividual reproducibility. We assessed SV using TTE, which has been validated against the thermodilution technique [30]. Thermodilution monitoring cannot be used because of recirculation phenomena [31], and preload reserve haemodynamic indices are not currently validated in this setting for the reasons described above. There were some concerns about the safety of PLR among ECMO patients. In our cohort, all patients underwent PLR without any adverse impact on the ECMO system. Before PLR, however, precautions were taken regarding the length of inflow and outflow cannulas.

## Conclusions

In this study, a > 10% increase in ΔPLRSV was predictive of fluid responsiveness in patients placed on venovenous ECMO respiratory assistance. This diagnostic procedure is easy to perform, reversible, familiar to intensive care physicians and easily reproducible, and it may be helpful in reliably identifying patients who will benefit from fluid loading. In contrast, we have not demonstrated that ΔPLRPO and ΔrespPP can be used to predict volume responsiveness. Further studies of a larger sample of patients placed on various types of ECMO are necessary to assess these results.

## Key messages

• Derivative pulse pressure indices (ΔrespPP and ΔPLRPP) failed to predict fluid responsiveness in ARDS patients placed on venovenous ECMO.

• A > 10% increase of ΔPLRSV may predict fluid responsiveness in patients treated with venovenous ECMO.

• ΔPLRPO cannot be used to predict fluid responsiveness.

## Abbreviations

ΔPLRPO: passive leg raising pump outflow change; ΔPLRPP: passive leg raising pulse pressure change; ΔPLRSV: passive leg raising stroke volume change; ΔrespPP: respiratory pulse pressure variation; ΔrespSV: respiratory stroke volume variation; ARDS: acute respiratory distress syndrome; CI_95_: 95% confidence interval; CO: cardiac output; CVC: central venous catheter; CVP: central venous pressure; DAP: diastolic arterial pressure; ECMO: extracorporeal membrane oxygenation; IRB: Institutional Review Board; LVEF: left ventricular ejection fraction; MAP: median arterial pressure; PI: pulse index; PO: pump outflow; PLR: passive leg raising; ROC: receiver operating characteristic; RPM: rotation per minute; SAP: systolic arterial pressure; SD: standard deviation; SV: stroke volume; TTE: transthoracic echocardiography; VE: volume expansion; VTIAo: aortic velocity-time integral ratio.

## Competing interests

The authors declare that they have no competing interests.

## Authors' contributions

PGG conceived, designed and coordinated the study and drafted the manuscript. EZ, MD and TC participated in the coordination of the study. MM, VH, LB and EB participated in the coordination of the study and helped in drafting the manuscript. HD performed the statistical analysis and helped in drafting the manuscript.
